# Immune escape of avian oncogenic Marek’s disease herpesvirus and antagonistic host immune responses

**DOI:** 10.1038/s41541-024-00905-0

**Published:** 2024-06-15

**Authors:** Zhi-Jian Zhu, Man Teng, Yu Liu, Fu-Jia Chen, Yongxiu Yao, En-Zhong Li, Jun Luo

**Affiliations:** 1 College of Biological and Food Engineering & Affiliated Central Hospital, Huanghuai University, Zhumadian, 463000 People’s Republic of China; 2 Institute for Animal Health & UK-China Center of Excellence for Research on Avian Disease, Henan Academy of Agricultural Sciences, Zhengzhou, 450002 People’s Republic of China; 3 Henan Provincial Key Laboratory of Animal Immunology, Henan Academy of Agricultural Sciences, Zhengzhou, 450002 People’s Republic of China; 4 Key Laboratory of Animal Immunology, Ministry of Agriculture and Rural Affairs of the People’s Republic of China, Zhengzhou, 450002 People’s Republic of China; 5 The Pirbright Institute & UK-China Centre of Excellence for Research on Avian Diseases, Pirbright, Ash Road, Guildford, Surrey GU24 0NF UK; 6 Laboratory of Functional Microbiology and Animal Health, College of Animal Science and Technology, Henan University of Science and Technology, Luoyang, 471003 People’s Republic of China; 7 Longhu Laboratory, Zhengzhou, 450046 People’s Republic of China

**Keywords:** Viral immune evasion, Viral infection

## Abstract

Marek’s disease virus (MDV) is a highly pathogenic and oncogenic alpha herpesvirus that causes Marek’s disease (MD), which is one of the most important immunosuppressive and rapid-onset neoplastic diseases in poultry. The onset of MD lymphomas and other clinical diseases can be efficiently prevented by vaccination; these vaccines are heralded as the first demonstration of a successful vaccination strategy against a cancer. However, the persistent evolution of epidemic MDV strains towards greater virulence has recently resulted in frequent outbreaks of MD in vaccinated chicken flocks worldwide. Herein, we provide an overall review focusing on the discovery and identification of the strategies by which MDV evades host immunity and attacks the immune system. We have also highlighted the decrease in the immune efficacy of current MD vaccines. The prospects, strategies and new techniques for the development of efficient MD vaccines, together with the possibilities of antiviral therapy in MD, are also discussed.

## Introduction

Marek’s disease (MD) is a highly contagious and rapidly progressive lymphoproliferative avian disease, that is caused by pathogenic Marek’s disease virus (MDV) and is characterized by severe immunosuppression, neurological disorders and malignant T-cell lymphomas^1^. In addition to the direct effects of the disease on mortality, MDV infection also causes secondary infections, decreased productivity, and increased morbidity. The causative agent of MD is gallid alphaherpesvirus 2 (GaAHV-2), which is also known as MDV type 1 (MDV-1), a prototypic member of the genus *Mardivirus* of the *Alphaherpesvirinae* subfamily that includes other associated avian herpesviruses, such as gallid alphaherpesvirus 3 (GaAHV-3)/MDV type 2 (MDV-2) and meleagrid alphaherpesvirus 1 (MeAHV-1)/turkey herpesvirus (HVT)^2^. Although they are antigenically related to MDV-1, MDV-2 and HVT are both non-pathogenic viruses in chickens. Based on their virulence and pathogenicity to birds, MDV-1 isolates can be further divided into distinct pathotypes, including mild (m), virulent (v), very virulent (vv) and very virulent plus (vv + ) MDVs^3^. As the first tumour disease that can be prevented by vaccination, MD has been well controlled in the 21^st^ century through large-scale vaccination programs^4^. However, the evolution of epidemic MDVs towards higher virulence, especially the newly emerged vv+MDV and hypervirulent MDV (HV-MDV) variants driven by long-term MD vaccination and imperfect immunization, has recently caused frequent outbreaks of MD in vaccinated chicken flocks worldwide^5–9^.

Since the 1970s, three widely used MD vaccines, including the FC-126 strain of HVT, the SB-1 strain of MDV-2, and the CVI988/Rispens (CVI988) strain of MDV-1, have been developed for the efficient control of disease^10^. Over the past several decades, CVI988 has been regarded as the most effective vaccine against MDV, but the underlying mechanisms that improve protective immunity following vaccination are not fully understood. Along with the persistent evolution of viruses, specific changes in MDV genomes have been documented, possibly contributing to the increase in virulence and allowing the virus to overcome MD vaccine protection^9,11–14^. Unfortunately, to date, there are few alternative vaccines, aside from CVI988, that can be used to combat the emerging HV-MDV variants^5,15^.

**Fig. 1:**
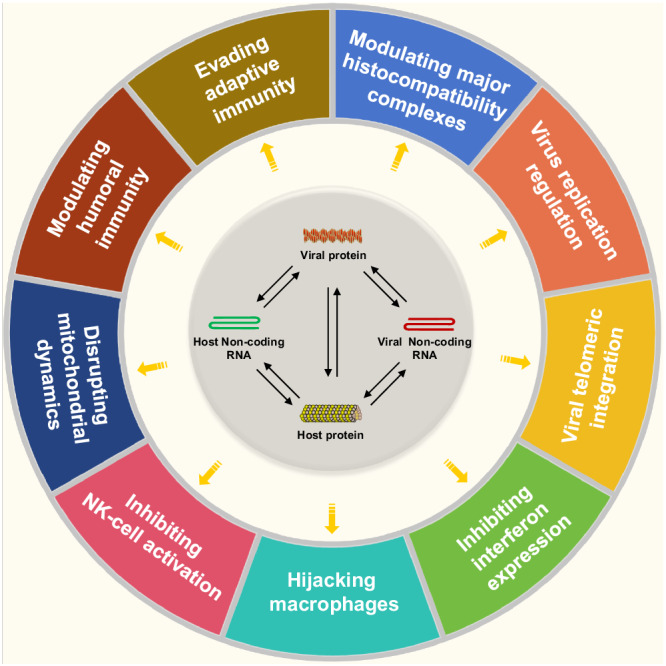
Schematic of the approaches by which MDV evades host surveillance and the immune response. The key strategies and potential approaches, such as modulation of histocompatibility complexes, regulation of virus replication, viral telomeric integration, inhibition of interferon expression, manipulation of macrophages, suppression of NK-cell activation, disruption of mitochondrial dynamics, modulation of humoral immunity, and evasion of adaptive immunity, are drawn together to demonstrate the intricate interactions between viral components, host cellular proteins, and/or non-coding RNAs to counteract the host immune response. (Created with Microsoft PowerPoint).

MDV invades host immune cells such as macrophages and lymphocytes^16,17^, leading to severe immunosuppression and resulting in weaker protection mediated by MD vaccination. As summarized in Fig. 1, MDV can evade host surveillance and immune responses via several mechanisms to provide a favourable environment for self-replication and proliferation. For example, MDV reduces the expression of viral immunogenic antigens through semi-productive cytolytic replication^18^ and downregulates the expression of major histocompatibility complex class I (MHC-I) on infected lymphocytes to achieve immune evasion^19^. The regulation of both viral and host cellular genes involved in immunity during MDV infection occurs at both the transcriptional and posttranslational levels to limit host immune responses to the virus^20,21^. MDV also encodes proteins that interfere with and modulate the host immune system, thus allowing viruses to establish lifelong persistent infection^22^. In addition to viral proteins, noncoding RNA genes (ncRNAs) are also involved in the MDV lifecycle, oncogenesis, and even immunoregulation^23^. In particular, some MDV-1-encoded microRNAs (miRNAs) recognize both viral and/or cellular genes to evade host innate and acquired immune mechanisms through epigenetic control and/or posttranslational regulation of mRNA targets. As one of the most virulent oncogenic herpesviruses, MDV has mastered the exploitation and modulation of host immune function. However, MDV-induced immunosuppression is very complex, poorly understood, and in many cases unrevealed. An overall review of the mechanisms of immune evasion and strategies of MDV evasion of the host immune system, together with a glance at the immune efficacy of widely used MD vaccines and the new generation of gene-editing technology, will be meaningful for the future development of novel MD vaccines and the efficient control of this disease.

## Host immune responses to MDV infection

MDV infection induces a comprehensive host immune response that includes the innate and acquired immune responses; this response includes cytokine release, immune cell recruitment, and changes in the expression patterns of host immune-related genes. The degree of immune response induced by the virus is quite different in hosts with different levels of MD resistance/susceptibility. Transcriptome analysis of bone marrow-derived macrophages and dendritic cells from susceptible or resistant chickens infected with MDV revealed a higher viral infection rate and stronger activation of the immune system in susceptible birds^24,25^, indicating that the development of MD resistance likely occurs during the early stage of infection through the innate immune response. Increased expression of key immune genes was detected in the innate immune cells from the resistant chickens compared to those from the susceptible chickens, thus showing inherent immune supremacy. However, after MDV challenge, only a limited number of immune-related genes, such as iNOS, which is thought to inhibit MDV replication by releasing NO, were highly expressed in the innate immune cells of the resistant chickens. Conversely, the expression of a number of genes involved in immune pathways, such as TLR and JAK-STAT signalling, were downregulated in the resistant chickens. On the one hand, this may be attributed to virus evasion of immune detection, consistent with the idea that MDV induces a latent phase in resistant birds. On the other hand, this approach could represent a defensive strategy employed by resistant hosts to disrupt the actin-mediated propagation of MDV between cells through the inactivation of immune pathways, thereby limiting MDV infection.

MDV also regulates the expression patterns of host immune-related genes at different stages of infection to facilitate pathological progression. A previous study showed that during the latent stage, vv+MDV induced measurable suppression of gene expression associated with host defence, but this suppression was followed by apparent activation of the defence response during viral reactivation in susceptible chickens^21^. Functional enrichment analysis of MDV-induced differentially expressed genes (DEGs) revealed that at 10 days post infection (dpi), a large number of genes that had downregulated expression in the susceptible line 7_2_ were significantly enriched in several terms associated with fatty acid metabolism, which may suppress virus replication and control cancer cell proliferation to play a role in establishing latency or repressing MDV infection. Only a few genes that had upregulated expression were enriched in two GO terms, immune response and immune effector process. However, at 21 dpi, 85% of the DEGs had upregulated expression and were significantly enriched in immune response-related terms, implying that a strong defence response was activated and ready to control tumorigenesis and disease progression during the reactivation stage. Interestingly, the expression of the immune factors IFN-α and IFN-β in the thymus and bursa of Fabricius was downregulated during the cytolytic infection and reactivation stages of MDV infection, but not during the latent stage^26^.

Notably, the differential expression of immune-related genes may also be related to the virulence and pathogenicity of distinct MDV strains. Using RNA sequencing (RNA-seq), transcriptomes of bursa and spleen lymphocytes from CVI988- or vvRB1B-challenged chickens preliminarily revealed differential immune responses initiated by vaccine or virulent MDV strains during early cytolytic infection^27^. The identification of highly significant genes associated with host immune competence showed that lymphocytes from CVI988-vaccinated chickens could elicit a stronger early immune response than lymphocytes from vvRB1B-infected chickens. Notably, the expression of IL-1β, IL-6, IL8L1, CCL4 and CCL5 was significantly upregulated in splenic lymphocytes from CVI988-infected birds compared to those from vvRB1B-infected birds; this experiment provided valuable data on the transcriptional landscape to understand vaccine-mediated immune protection mechanism.

## Immune evasion during different phases of virus infection

### Modulation of major histocompatibility complexes

Generally, herpesviruses can modulate the expression of both MHC-I and MHC-II molecules in infected immune cells, such as macrophages and B cells, to evade host cell-mediated immunity. The MHC gene family encodes immunity-related cell surface molecules that are primarily involved in antigen presentation and the regulation of T lymphocytes and impact the specificity of the immune response to virus- and MD-transformed cells. Chicken MHC haplotypes differ in their ability to present various kinds of peptides due to MHC-peptide interactions, and these differences may be responsible for MHC-related resistance to MD^28^. All MDV serotypes are reported to reduce the surface expression of MHC-I glycoproteins during viral lytic replication, which is believed to facilitate immune evasion by the virus^19^. Further studies have demonstrated that two MDV genes, MDV012 and pUL.49.5, which are listed in Table 1, play a role in the downregulation of MHC-I molecule expression by interfering with the function of transporters associated with antigen presentation (TAPs)^29,30^. Compared to those of the MHC-I system, few studies have investigated chicken MHC-II genes and molecules. The vast majority of MDV peptide epitopes presented by chicken MHC-II molecules arise from only four viral proteins, namely, glycoproteins gH, gI, and gE and the UL43 tegument protein^31^. MDV infection-related up- or downregulation of MHC-II expression depends on both the genetic background of the chicken host and the strain of virus^32,33^. The downregulation of MHC-II expression caused by MDV infection could be easily associated with viral immune escape, but the role of the upregulation of MHC-II expression in the immunopathological response needs to be further investigated.

**Table 1:** Targets and functions of the viral components involved in immune evasion during MD infection

Category	Viral components	Viral/host targets	Potential role and functions	Refs.
Modulation of MHC	MDV012	TAP	downregulation of MHC-I molecules expressions	^29^
	pUL.49.5	TAP	downregulation of MHC-I molecules expressions	^30^
	UD	UD	upregulation of MHC-II molecules expressions	^32^
	UD	IFN-γ-receptor complex	downregulation of MHC-II molecules expressions	^33^
Promote latency	meq	Bcl-2	inhibits infected cells apoptosis	^34^
		pp38-pp24	prevents viral DNA replication and productive infection	^35^
	vTR	chTERT	inhibits infected cells apoptosis	^38^
	miR-M7-5p	ICP4/ICP27	prevents viral DNA replication	^39^
	miR-M4-5p	UL28	prevents viral DNA replication	^42^
		JARID2	promote infected cells survival	^71^
	miR-M4-3p	UL32	prevents viral DNA replication	^42^
	miR-M5-3p	ICP22	prevents viral DNA replication	^40^
	UD	IKFZ1	maintenance of the latency	^44^
Telomeric integration	mTMR	/	facilitates viral genome integration	^48^
	sTMR	/	facilitates viral genome integration	^49^
	vTR	chTERT	participates in MDV integration	^37^
	Meq	c-Myc	participates in MDV integration	^51,52^
	VP22	UD	induces DNA lesions	^54,55^

*MHC* Major histocompatibility complex, *MDV012* MDV gene MDV012, *TAP* Transporter associated with antigen presentation, pUL.49.5, MDV UL49.5 gene, *Meq* MDV EcoRI-Q, *Bcl-2* B-cell lymphoma-2, *pp38* phosphoprotein 38, *pp24* Phosphoprotein 24, *vTR* Viral telomerase RNA, *chTERT* Chicken telomerase reverse transcriptase, *ICP4* Infected cell protein 4, *ICP27* Infected cell protein 27, *UL28* Unique long gene 28, *JARID2* Jumonji, AT rich interactive domain 2, *UL32* Unique long gene 32, *ICP22* Infected cell protein 22, *IKFZ1* Ikaros, *mTMR* Multiple telomeric repeats, *sTMR* Short telomeric repeats, *c-Myc* MYC proto-oncogene, *VP22* capsid protein VP22.

### Self-inhibition of viral activity during latency

The presence of latent MDV particles in host cells usually occurs at approximately 7 dpi, primarily in CD4^+^ T lymphocytes of virus-challenged birds. During the latent phase, expression of the viral genome is limited to only some specific genes that are required to maintain latency, whereas no progeny virions are produced, and the latent virus effectively evades host immune system surveillance^18^. As listed in Table 1, the currently known MDV latency-related genes located in the long and short repeat regions of the viral genome include the major oncogene *meq*, the viral telomerase RNA (vTR), latency-associated transcripts (LATs) and a number of viral miRNAs.

The *meq* gene encodes a basic leucine zipper transcription factor with favoured dimerization with the oncoprotein c-Jun (Meq/c-Jun) and itself (Meq/Meq). The binding of Meq/c-Jun to AP-1-containing promoters facilitates the survival of latently infected cells through activation of the expression of the antiapoptotic factor Bcl-2^34^. Binding of the homodimer Meq/Meq to the MERE II site of the MDV replication origin suppresses transcription from flanking bidirectional promoters (*pp38*/*24* and *pp14*)^35^, thus potentially impeding the expression of early lytic genes, such as pp38. This transcriptional regulation may hinder viral replication and productive infection; thus, this regulation warrants further investigation. Meq may also help maintain latent MDV infection in combination with other transcription factors through the modification of host and viral gene expression^20,36^. MDV is the only virus known to encode a telomerase RNA subunit (vTR), and MDV vTR shares 88% sequence identity with chicken telomerase RNA (chTR). vTR mainly serves as a template for the addition of telomeric repeats and interacts with chicken telomerase reverse transcriptase (TERT) to participate in MDV integration^37^. Moreover, vTR has also been speculated to have antiapoptotic effects on latently infected cells^37,38^. LATs are a family of long ncRNAs (~10 kb) that are expressed mainly during virus latency and lymphomagenesis, suggesting that they play a role in the maintenance of latency and/or cell transformation. Small ncRNAs, such as MDV-1 miRNAs, have also been demonstrated to target both viral and cellular mRNAs to promote long-term persistent infection. For example, miR-M7-5p has been verified to target the MDV-1 immediate-early genes ICP4 and ICP27, thus contributing to the establishment and maintenance of latency^39^. miR-M5-3p, another MDV-1 miRNA, is also reported to downregulate the expression of viral infected cell protein 22 (ICP22), which is required for the lytic replication of viruses^40^. miR-M4-5p, a viral orthologue of cellular oncogenic miR-155 that plays a crucial role in MDV tumorigenesis, can directly modulate the expression of viral targets such as UL28 and UL32^41,42^. Thus, maintaining viral latency has also been suggested as a potential mechanism for miR-M4-5p^43^.

In addition to viral genes, some host proteins may be hijacked to help establish MDV latency. Ikaros (IKFZ1) has recently been validated as a cancer driver gene for MD lymphomas^44^, and it is speculated to be involved in latent MDV infection because of its important role in regulation of the herpesvirus latent-lytic switch^45^.

### Telomeric integration and MDV immune escape

In latently infected cells and tumour cells, MDV specifically integrates its genome in the telomeric region of host chromosomes. MDVs may use integration not only to avoid host immunosurveillance and maintain latency but also to maintain persistence in host chromosomes, viral genome replication, and host cell proliferation^46^. Furthermore, telomeres repress the expression of adjacent genes, which is beneficial for silencing the virus genome during the establishment of latency. As listed in Table 1, there is a growing amount of data on the viral components involved in the integration process, but the exact mechanism and factors required for MDV integration are not fully understood^47^.

MDV harbours two viral telomeric repeat (TMR) arrays, multiple telomeric repeats (mTMR) and short telomeric repeats (sTMR), which are identical to the host telomere sequence of TTAGGG. The viral TMR is hypothesized to serve as a mini-chromosome cap or contribute to the ability of the viral genome to integrate into the host genome via recombination or a telomerase-based pathway. Both mTMR and sTMR have been shown to facilitate integration of the MDV genome into host telomeres during latency^48,49^. The expression of vTR is regulated by DNA methylation patterns in functional c-Myc response elements (REs) of the vTR promoter, thereby controlling MDV integration through vTR-mediated telomerase activity^50,51^. The absence of a cell population exhibiting a telomere-integrated phenotype following infection with the Δ*meq* MDV strain suggests that the oncogene *meq* plays a role in facilitating telomeric integration of MDV in activated lymphocytes^52^. Considering the transcriptional regulation of vTR by c-Myc through the functional c-Myc RE within the vTR promoter, we speculate that Meq indirectly regulates vTR through c-Myc expression, which is induced by Meq/c-Jun heterodimers, thereby enhancing the function of viral telomere integration^34,51^. miR-M4-5p may also participate in MDV integration as it has been demonstrated to target the TGF-β signalling pathway by downregulating LTBP1 expression to activate the expression of c-Myc^53^. In addition, the VP22-induced DNA lesions observed at the early stage of virus infection in vivo may facilitate MDV integration directly or indirectly by triggering the DNA damage response (DDR) and DNA repair pathways, especially homologous recombination^54,55^. Recently, based on a quantitative integration assay using chicken T-cell lines as targets for MDV latency and transformation, a novel cell culture system for in vitro integration has been developed to provide a tool for revealing the mechanism of MDV integration into host telomeres in the future^56^.

## Immune damage and immunosuppression of the host immune system

MDV induces serious immunosuppression (IS) through a variety of mechanisms during pathogenesis, and IS is often divided into two phases: an early IS phase associated with early cytolytic infection of lymphoid organs and a late IS phase associated with the reactivation of MDV and the development of tumours^57^. The stage of MDV-induced IS involves the viral component-mediated regulation of host innate and adaptive immune responses (Table 2).

**Table 2:** Targets and potential functions of the viral components involved in immunosuppression of the host immune system

Category	Viral components	Viral/host targets	Potential role and functions	Refs.
Modulation of innate immunity	Meq	STING	prevents IRF7 activation and IFN-β induction	^20^
		CD107 (LAMP-1)	influences NK cell activation	^83^
	RLORF4	p65/p50	blocks IFN-β promoter activation	^60^
	VP23	IRF7	blocks the nuclear translocation of IRF7	^61^
	US3	IRF7	blocks the nuclear translocation of IRF7	^62^
		CREB	regulates the transcription function of the CREB/Meq	^63^
	UD	DDX5	blocks TLR3 signalling cascades	^66^
	miR-M4-5p	ADAR1	inhibits the activity of MDA5	^67^
		TLR3	regulates TLR3-mediated signalling pathway	^70^
	UD	MIF	sustains macrophage survival	^76–78^
			exerts significant pro-tumour effects	
	UD	BF1/BF2	inhibits NK cell activation	^82^
	vNr-13	UD	disrupted mitochondrial network morphology	^87^
	UD	POLG2	induces imbalance of mitochondrial contents and gene expression	^88^
Modulation to adaptive immunity	pp38	UD	results in B-cell apoptosis, atrophy of lymphoid organs	^17^
	UD	anti-senescence factors	prolongs survival of infected B cells	^92^
	UD	PD-1	keeps activated T cells from killing tumour cells or infected cells	^95,96^
	UD	IL-10/CTLA-4	induces immunosuppressive effect of Treg cells	^95,98^
	vIL-8	UD	recruits Treg cells (CD4^+^ CD25^+^ T cells)	^99^
	UD	COX-2/PGE2	impairs T-Cell Proliferation	^110^

*STING* Stimulator of interferon genes, *p65/p50* NF-kappa B p65/p50 subunit, *VP23* capsid protein VP23, *US3* Unique-short kinase 3, *CREB* cAMP response element-binding protein, *DDX5* Dead-box Helicase 5, *ADAR1* Adenosine deaminase acting on RNA1, *TLR3* Toll-like receptor 3, *MIF* Macrophage migration inhibitory factor, *BF1/BF2* The chicken’s major histocompatibility complex class I major (BF2) and minor (BF1) glycoproteins, *CD107* (LAMP-1) Lysosome-associated membrane protein 1, *vNr-13* Viral anti-apoptotic protein Nr-13, *pp38* Phosphoprotein 38, *PD-1* Programmed cell death 1, *IL-10* interleukin-10, *CTLA-4* cytotoxic T lymphocyte antigen 4, *vIL-8* Viral interleukin-8, *COX-2* Cyclooxygenase-2, *PGE2* Production of prostaglandin E2.

*UD*, undefined.

### Viral strategies to combat innate immunity

Upon viral infection, host cells recognize pathogenic microorganisms through pattern recognition receptors (PRRs) to activate the first defence system, namely, the innate immune system, to eliminate pathogenic microorganisms or activate the adaptive immune response. As a smart pathogen, MDV has also evolved various strategies to evade the innate immune system.

#### Interferons (IFNs)

**Fig. 2:**
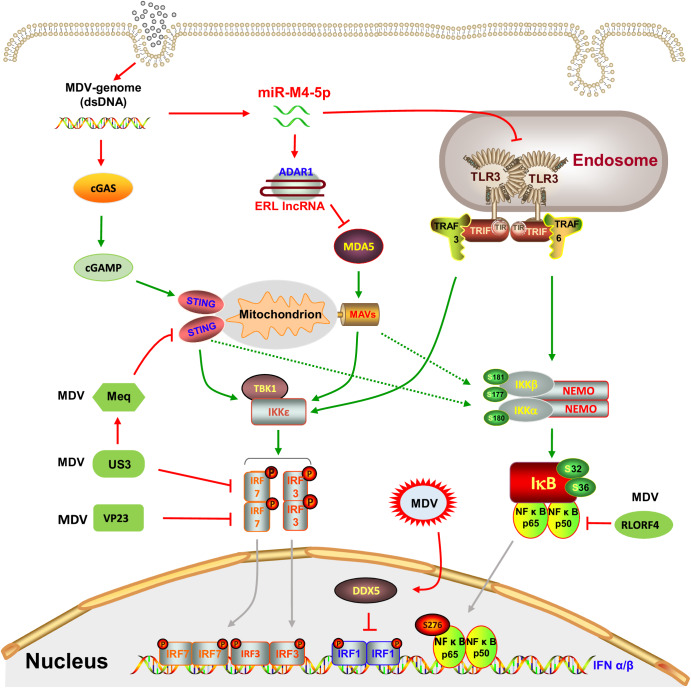
Schematic diagram of the mechanism by which MDV evades PRR-mediated type I interferon signaling pathways during viral infection. MDVs exploit viral proteins and noncoding RNAs to evade host innate immunity by inhibiting the cGAS-STING signaling pathway, the MDA5-mediated signaling pathway and the Toll-like receptor signaling pathway. cGAS, cyclic GMP-AMP synthase; cGAMP, cyclic GMP-AMP; STING, stimulator of interferon genes; MDA5, melanoma-differentiation-associated gene 5; MAVS, mitochondrial antiviral signaling protein; TLR3, Toll-like receptor 3; TRIF, TIR-domain-containing adapter inducing interferon-β; TRAF, tumor necrosis factor receptor associated factor; TBK1, TANK-binding kinase 1; IKKε, nuclear factor kappa-B kinase epsilon; IRF, interferon regulatory transcription factor; NF-κB, nuclear factor-kappa B; NEMO, nuclear factor (NF)-kappa B essential modulator; IKKα, NF-kappa B kinase subunit alpha; IKKβ, nuclear factor kappa-B kinase beta; IκBα/β, nuclear factor kappa-B-α/β; ADAR1, adenosine deaminase acting on RNA1; DDX5, Dead-box Helicase 5. (Created with Microsoft PowerPoint).

IFNs are widely expressed cytokines that possess strong antiviral and immunomodulatory properties and are mainly classified into three types: type I (IFN-α and IFN-β), type II (IFN-γ) and type III (IFN-λ). The role of IFNs in MDV replication has been confirmed through both in vitro and in vivo models, in which IFNs not only reduce the plaque formation and expression of pp38 and gB in virus-infected cells but also significantly delay the onset and progression of disease^58^. The protective role of IFNs is also indicated by the differential expression patterns of the IRF3 and IFN-β genes in resistant and susceptible chicken lines^59^. However, the expression of IFNs is significantly decreased in the thymus and bursa of Fabricius in MDV-challenged birds at the lytic infection stage, implying that MDV inhibits IFN-I expression in the host and results in immunosuppression^26^. One of the important strategies by which MDV evades the IFN-mediated innate immune response is to hijack host proteins involved in the IFN signal transduction pathway. cGMP-AMP synthase (cGAS), which is an important cytosolic DNA sensor, detects cytosolic viral DNA via stimulator of interferon genes (STING) to initiate the innate antiviral response, indicating that the cGAS-STING pathway plays an important role in the host response to MDV infection^20^. As demonstrated in Fig. 2, five MDV proteins, namely, Meq, RLORF4, US3, UL46 and VP23, reduce IFN-β induction by regulating the cGAS-STING pathway. Meq inhibits STING signalling by impairing the assembly of the STING-TBK1-IRF7 complex, thereby preventing IRF7 activation and the induction of IFN-β. Deletion of the transactivation domain of Meq may impact its immunosuppressive effects by disrupting the interaction between STING and the C-terminal domain of Meq, consequently leading to a reduction in viral pathogenicity^20^. RLORF4 was found to suppress nuclear translocation of the endogenous NF-κB subunits p65 and p50, which ultimately blocks IFN-β promoter activation induced by cGAS and STING^60^. The other two viral proteins, US3 and VP23, were both shown to suppress interferon stimulatory DNA (ISD)-triggered IFN-β production by blocking the nuclear translocation of IRF7 through different strategies^61,62^. As a multifunctional serine/threonine protein kinase, US3 is also involved in the regulation of various cellular pathways by regulating the transcriptional function of the CREB/Meq heterodimer in a phosphorylation-dependent manner, which influences cellular and viral gene expression^63^. In addition to functioning as cytosolic DNA sensors, Toll-like receptors (TLRs) also function in the induction of proinflammatory cytokine and IFN-β production in the host immune response to MDV infection^64,65^. Strikingly, MDV infection also triggers Dead-box Helicase 5 (DDX5) to thwart the activation of IFN-β by blocking Toll-like receptor 3 (TLR3) signalling cascades; this result provides new insight into the mechanism by which viruses hijack host proteins to achieve immunosuppression^66^.

MDV also encodes viral miRNAs or regulates host miRNAs to target host proteins and inhibit IFN-regulated host antiviral responses. miR-M4-5p has a wide range of effects on the cellular environment and the global gene expression of lymphocytes by targeting transcription factors such as PU.1, which plays an important role in immune regulation. miR-M4-5p enhances the expression of adenosine deaminase, which acts on RNA1 (ADAR1), thereby inducing hyper-editing of edited repeat long (ERL) lncRNA^67^. Hyper-edited ERL lncRNAs are hypothesized to disrupt the innate immune system by impeding the activity of MDA5, as the binding of inosine-containing RNAs by MDA-5 is known to inhibit activity and to block IFN expression^68,69^. Targeting and regulating the TLR3-mediated signalling pathway via miR-M4-5p is another way for MDV to inhibit the host innate immune response^70^. In addition, both miR-M2-3p and miR-M9-5p were predicted to target IL-18, which is a proinflammatory cytokine that stimulates IFN-γ production; this targeting may allow the virus to evade host defences^71^. Some cellular miRNAs, such as gga-miR-155, have also been shown to help viruses evade the host innate immune system^72^. Interestingly, MDV is known to replace and downregulate miR-155 expression in host cells through its own orthologue, miR-M4-5p, thereby exploiting the regulatory pathways of miR-155 by effectively modulating the targets associated with this miRNA^71,73^. Notably, viral miRNA-mediated inhibition of host miRNA processing represents a ubiquitous cellular mechanism by which herpesviruses disrupt intrinsic host immunity ^74^; this mechanism will be a very intriguing focus for future research on MDV pathogenesis.

#### Innate immune cells

Innate immune cells are composed mainly of phagocytes and nonphagocytic cells. Among them, macrophages and dendritic cells, as antigen-presenting cells (APCs) involved in the initiation of the immune response, play important roles in the development of immunity against MDV. The role of dendritic cells in MD immunology is quite limited, but they are thought to be involved in the initiation of host innate and adaptive immunity^25^. The role of macrophages in anti-MDV infection can be determined based on vaccination-activated macrophages and the increase in macrophage populations within the duodenum in MD-resistant and MD-susceptible chickens^75^. In addition to presenting MDV antigens in association with MHC class I and II molecules to initiate adaptive immunity, macrophages are also directly involved in the inhibition of MDV replication and disease development. Unfortunately, macrophages become infected either directly or after an initial round of MDV replication, which leads to macrophage death and heavy infiltration around blood vessels. The virus can also replicate in macrophages, making macrophages excellent candidates for transporting MDV to primary and secondary lymphoid organs. Moreover, macrophage migration inhibitory factor (MIF), a soluble mediator secreted by activated T cells that inhibits the migration of macrophages, was reported to be involved in MD pathogenesis^76^. On the one hand, decreased expression of MIF during early and latent infection enhances macrophage migration, which might be a potential mechanism by which MDV increases virus transport and spread. On the other hand, upregulation of MIF expression after latent infection might be employed by MDV to induce lymphoma occurrence. First, MIF sustains macrophage survival and function by suppressing p53-dependent apoptosis, which is important for MDV-infected macrophages to spread the virus^77^. Second, MIF exerts significant protumour effects by regulating antitumour T lymphocyte responses^78^. Compared to activated and fully functional macrophages, tumour-associated macrophages (TAMs) produce tumour growth-promoting factors and induce immunosuppression by releasing immunomodulatory factors^79^. Immunoregulatory macrophages isolated from MDV-infected chickens exhibit abilities similar to those of TAMs and are correlated with transient immunosuppression during the primary cytolytic phase of infection.

Natural killer (NK) cells are key players in the innate immune response and can respond to stimuli and produce antiviral cytokines such as IFN-γ. These cells recognize virus-infected cells via the ligation of cell death receptors and the release of granules. The various viral strategies used to modulate the recognition of herpesviruses indicate the importance of NK cells in the control of such viruses^80,81^. The NK cells in MDV-infected chickens are more active than those isolated from uninfected birds, and in resistant chickens, this activity lasts longer than that in susceptible chickens. However, the mechanisms of NK cell evasion by MDV have largely remained elusive. MDV reduced the expression of the MHC molecule BF2 and prevented the downregulation of BF1 expression; BF1 specifically interacts with NK cells, thereby inhibiting NK cell activation^82^. Recent data have also provided evidence that NK cells can be efficiently infected by MDV and that cell activation is dependent on the expression of the major oncogene *meq*, thus providing additional evidence for NK cell-related immune regulation mediated by MDV^83^.

#### Mitochondria

Mitochondria are crucial cellular organelles in eukaryotes and participate in many cell processes, including the immune response, growth development, and tumorigenesis. Viral infection can activate or inhibit mitochondrial function, alter mitochondrial contents, and influence gene expression. Mitochondrial DNA (mtDNA) is normally present at thousands of copies per cell and is packaged into several hundred higher-order structures termed nucleoids^84^, and mtDNA stress elicited by herpesviruses engages the DNA sensor cGAS and promotes STING-IRF3-dependent signalling to increase ISG expression, potentiate IFN-I responses, and confer viral resistance^85^. Moreover, *alphaherpesviruses* have evolved mechanisms to disrupt mitochondrial dynamics, cause mitochondrial dysfunction and enhance viral infection and pathogenesis^86^. HVT-encoded vNr-13, the first known *alphaherpesvirus*-encoded Bcl-2 homologue of Nr-13, localizes to the mitochondria and endoplasmic reticulum (ER) and disrupts mitochondrial network morphology in virus-infected cells to modulate the host immune response^87^. MDV infection results in an imbalance in mitochondrial content and gene expression, especially significantly reduced levels of mtDNA in the spleens of MD-susceptible 7_2_ birds, suggesting that mitochondrial dysfunction is indispensable for virally induced cell transformation and subsequent disease development in chickens^88^. MDV-induced mtDNA stress is necessary for effective ISG expression and antiviral priming, but mtDNA damage can also lead to inflammation and apoptosis and ultimately trigger oncogenesis in chickens.

### Viral strategies to combat adaptive immunity

The key components of adaptive immunity are B and T lymphocytes, which are involved in tightly regulated interactions with antigen-presenting cells and facilitate pathogen-specific immunologic effector pathways, generate immunologic memory, and regulate host immune homeostasis. B cells are involved in the humoral immune response, whereas T cells are involved in the cell-mediated immune response. The early pathogenesis of MDV is characterized by a burst of productive/restrictive infection in B cells, followed by a latent infection in activated CD4^+^ T cells that can persist for three weeks prior to reactivation and transformation^38^. Destruction of B and T cells during lytic infection ultimately leads to severe atrophy of immune organs and consequential immunosuppression in hosts^17^.

#### Humoral immunity

Due to the strict cell-associated nature of MDV, B-cell-mediated humoral immunity may be limited to provide protection against the virus. However, various studies on host responses to MDV infection in the bursa have shown that the complete removal of B cells by surgical bursectomy strongly influences MDV-induced pathogenesis and reduces vaccine-mediated immune protection^89^. The role of humoral immunity in controlling MDV infection has also been confirmed by the presence of maternal antibodies that delay the development of clinical signs and tumours^38^. However, recent research has shown that B cells do not play an essential role in vaccine-mediated immunity in response to oncogenic MDV strains^90^. Although the antiviral capacity of B cells during MDV infection needs to be further investigated, it has been well documented that B cells are hijacked by MDV to promote the development of disease. B cells are the primary target cells by which MDV is transported to secondary lymphoid tissues after phagocytosis by macrophages, resulting in early cytolytic infection in the lungs. The lytic activity of MDV-1 pp38 in B cells results in cell apoptosis, the atrophy of lymphoid organs, and a significant decrease in antibody production and antiviral defence, which ultimately causes host immunosuppression^17^. The route by which MDV particles spread from infected B cells to activated T cells and different organs leading to systemic infection should not be overlooked, although the presence of B cells acting as the initial targets of MDV infection has recently been verified to not be an essential step in the activation and consequent infection of a larger number of CD4^+^ T cells^90,91^. Interestingly, in addition to inducing the apoptosis of uninfected B cells, leading to immunosuppression, MDV prolongs the survival of infected B cells by inducing a senescence-like phenotype, which may promote the persistence of viruses and thus allow more time to recruit and infect T cells^92^. Strikingly, MHC-II molecules, which are embedded in the membranes of APCs such as macrophages, dendritic cells, and B cells, play a crucial role in activating B-cell proliferation and differentiation^93^. The modulation of MHC-II by MDV mentioned above might contribute to the limitations in humoral immunity during MDV infection and/or vaccine-induced protection. Taken together, these findings indicate that MDV hijacks B cells in different ways, not only by using them as carriers for virus transmission and diffusion but also by limiting the humoral immune response of the host.

#### Cell-mediated immunity

MDV is a strict cell-associated herpesvirus, and its transmission in vivo depends on cell-to-cell contact; thus, T-cell-mediated immunity is thought to play a more crucial role than the antibody-mediated response. Both antiviral and antitumour T-cell immunity are induced by MDV or MD lymphoblastoid cells to suppress viral replication and prevent the development of tumours, respectively^94^. While T-cell-mediated immune responses play a pivotal role in combating viral infections, MDV has evolved and developed sophisticated mechanisms for the suppression and evasion of cellular and soluble effectors of the adaptive immune system.

T-helper cells (CD4^+^) not only act as a bridge between specific APCs and B cells and CD8^+^ T cells but also help these cells through interactions between CD40 and CD40L, thereby promoting the activation of cytotoxic CD8^+^ T cells and facilitating the survival, proliferation, and immunoglobulin class switching of B cells. MDV transforms CD4^+^ T cells approximately three weeks post infection and subsequently leads to atrophy of the thymus, the apoptosis of infected T cells and the development of MD lymphomas^38^. In addition, the expression of PD-1 was increased on CD4^+^ T cells of virus-infected birds at 21 dpi^95^, which prevented T cells from killing tumour cells or infected target cells in the setting of persistent infection and cancer^96^. Compared to those in CD4^+^ T cells in the control group, MDV altered the ubiquitylome associated with signal transduction and the immune system, cancer, and infectious disease pathways in CD4^+^ T lymphoma cells; this result may facilitate future studies on the mechanisms underlying MDV-induced immunosuppression and tumorigenesis in T cells in relation to the regulation of ubiquitination^97^.

Regulatory T cells (Tregs) are a subset of CD4^+^ T cells that exert immunoregulatory effects via the secretion of immunosuppressive soluble factors such as IL-10 and TGF-β, and cell contact-mediated regulation through costimulatory molecules such as cytotoxic T lymphocyte antigen 4 (CTLA-4). The immunosuppressive role of Treg cells has been indicated by the increased expression of IL-10 and CTLA-4 in CD4^+^ T cells during MDV infection, and this effect was more pronounced in MDV-susceptible chickens^95,98^. The MDV-encoded viral IL-8 (vIL-8) exhibits specific binding affinity for Treg cells (CD4^+^CD25^+^ T cells), potentially facilitating their recruitment as a target for MDV infection and subsequent transformation^99^. In addition, a novel subset of Treg cells expressing TGF-β on the surface was identified in different lymphoid tissues. The frequency of this population was greater in the spleens of MDV-susceptible chicken lines than in those of the resistant line, suggesting that this population plays an important role in MDV pathogenesis and immunosuppression^100^. Recently, the presence of CD4^+^CD25^+^ and CD4^+^ TGF-β^+^ Treg cells in the feathers of infected chickens has also demonstrated the ability of MDV to induce an immunomodulatory microenvironment in chicken feathers to achieve immune escape^101^.

Given that CD4^+^ cells are primarily hijacked by MDV to promote disease development and tumour formation, other populations of T cells, such as CD8^+^ and γδ T cells, may play crucial roles in the immune response to MDV infection. The important role of CD8^+^ T cells has been demonstrated by depleting CD8^+^ cells using monoclonal antibodies, which increased the likelihood of tumour occurrence and decreased vaccine immune protection against MD^102^. Subsequent studies have revealed a direct relationship between the magnitude of the T-cell response to MDV-1 antigens (pp38 and Meq) and host resistance to disease, which are elicited differently in MD-resistant and susceptible chickens^103^. Moreover, the potential role of γδ T cells was characterized by their significant increase in the spleen but decrease in the caecal tonsils during MDV infection, accompanied by the upregulation of IFN-γ expression in the early stage of infection and the upregulation of IL-10 expression during the late phases^104,105^. In addition, γδ T cells, similar to CD8^+^ T cells, play a critical role in early immune protection conferred by the CVI988 vaccine through differentially expressed cytotoxic and T-cell-related cytokines, except that CVI988 induces memory CD8^+^ T cells but not memory γδ T cells in chickens^106,107^. Unfortunately, persistent infection caused by MDV infection or vaccination may lead to T-cell exhaustion, which is characterized by the progressive loss of T-cell effector functions and memory properties and the upregulated expression of inhibitory receptors such as PD-1 and CTLA-4 on T cells^108^. Indeed, MDV infection upregulates the expression of CTLA-4, PD-1, and PD-L1 in infected chickens^95,109^. Furthermore, MDV infection also induces the activation of cyclooxygenase-2 (COX-2) and the production of prostaglandin E2 (PGE2), which contributes to the impairment of T-cell proliferation^110^.

## Imperfect MD vaccination and immune failure

In most cases, MD vaccination can prevent the development of tumours but cannot block persistent MDV infection and transmission. At present, little is known about the mechanism of MD vaccine-induced immunity. Early studies suggested that vaccine-induced adaptive immunity may play a role in providing protection against MD. Multiple MDV antigens, e.g., gB, once administered as a recombinant vaccine, are immunogenic and capable of triggering immune protection. However, the types and magnitude of vaccine-induced protective immune responses to these viral antigens are still unknown. Recent studies have cast doubt on the role of T cells in MD vaccine-mediated immunity against the development of virus-induced tumours, and this doubt has made the molecular mechanism of vaccine-induced protection even more elusive^111^. However, compared to those in previous studies, the differences in the role of T cells in MD vaccination between distinct studies might be due to differences in immune efficacy or the mechanisms of the MD vaccines^102,111,112^. Moreover, several other factors, including the genetic background of chickens^103^, the presence or absence of maternal antibodies, the virulence of MDV epidemic strains, vaccination doses and coinfections with other immunosuppressive and neoplastic pathogens, can also affect the immune efficacy of MD vaccines^113^.

Since the first introduction of the MD vaccine in the 1970s, MDV has overcome the vaccine barrier through the acquisition of numerous genomic mutations, and this evolutionarily adapted virus is more lethal to chicken hosts. Widely used MD vaccines involving more virulent MDV strains have been developed, suggesting that vaccination might be a key driver of the increase in virulence^114^. First, MD vaccines protect chickens against the disease but still allow virus infection and transmission, which causes vvMDV epidemic strains to circulate in vaccinated chickens and potentially increases the virulence of the circulating virus by providing selective pressure^115^. Second, the use of live attenuated MD vaccines also has potential adverse effects, including the reversion of pathogenicity, recombination, and functional complementation in the host. Concerns about use of MD vaccine recombination to create virulent viruses have increased. In recent studies^116,117^, several MDV strains generated via recombination of the CVI988 backbone and the partial unique short region of the virulent MDV strain have been isolated and characterized from vaccinated chicken flocks; these strains confirm the role of live MD vaccines as genetic donors for viral genomic recombination. Complementation is another adverse mechanism associated with MD vaccines. Using natural coinfection models, the MD vaccine and MDV have been shown to infect the same cells in vivo, resulting in functional complementation, and the nontransmissible MDV was transmitted to chickens via one round of transmission^118^.

Due to the emergence of vv+MDV strains and HV-MDV variants, developing novel and robust next-generation MD vaccines is necessary. One attractive strategy for producing novel effective vaccine strains is to use a recombinant MDV candidate attenuated by the deletion of *meq*, which completely disables tumour induction and provides superior protection against MDV epidemic strains with hypervirulence. However, *meq*-deleted MDV mutants usually retain the ability to induce significant lymphoid organ atrophy, which has been the main obstacle for their use as vaccine candidates. A cell culture passage-attenuated *meq*-null MDV strain was generated to avoid this disadvantage, but this strain still showed a slight immunosuppressive effect^119^. In recent years, MDV mutants with double deletions of *meq* plus a viral early cytolytic replication gene, such as *vIL8*, *pp38* and thymidine kinase (*tk*), have been generated to overcome this problem^120–122^. However, the protective efficacy of these vaccine candidates was significantly lower than that of the MDVΔMeq and CVI988/Rispens viruses. Considering that some ncRNAs, especially viral miRNAs, are also involved in the induction of lymphoid organ atrophy^123,124^, considering the deletion of MDV-miRNAs to reduce virus immunosuppression and improve the safety of *meq*-mutated MDV strains as vaccines might be worthwhile.

## Discussion

Most MDV genes play important roles in DNA replication, particle formation, immunosuppression, and many other processes that are essential in the viral lifecycle. The lack of sufficient immunological techniques and key reagents makes determining the dynamic changes and functional regulation of the host immune system during MDV infection a challenge. In future studies, three pivotal tasks involved in immune regulation in response to MDV infection need to be addressed, including determining which major viral components are involved in regulating host immune responses, how MDV genes or proteins dynamically respond to the immune system to achieve immunosuppression, and how to develop a more perfect MD vaccine for disease control in the future.

Currently, a comprehensive understanding of the effector molecules expressed by MDV after virus infection or vaccination is lacking. The best characterized gene is the major oncogene *meq*, which is crucial for MDV pathogenesis as it not only regulates tumorigenesis but also controls the host immune response. The current “gold standard” MD vaccine strain, which is live-attenuated CVI988, expresses two *meq* isoforms, which are designated small *meq* (*S-meq*) with a regular size and long *meq* (*L-meq*) harbouring an 180 bp insertion in the transactivation domain. *L-meq* strongly enhances viral pathogenesis and tumorigenesis, while *S-meq* completely abrogates pathogenesis, indicating that changes in the *meq* gene and other mutations in the CVI988 genome may contribute to virus attenuation^125^. Recently, studies have shown that both gene insertion in *L-meq* and deletion in *S-meq* can enhance the transactivation of Meq, but insertion also increases the pathogenicity of MDV, while deletion reduces its pathogenicity^14^. Additionally, minor polymorphisms in *meq* acquired during evolution allow the virus to overcome innate cellular responses and vaccination-induced protection^126^. To better understand the molecular basis of MDV pathogenesis and enhanced virulence, further investigating the functions of viral proteins, including Meq, is necessary. In addition, MDV encodes a series of ncRNAs that are crucial for immune regulation, including miRNAs, lncRNAs, and circRNAs^23^. In particular, the immunoregulatory effects of MDV-encoded miRNAs have been well characterized. Considering the impact of viral miRNA-mediated inhibition on host miRNA processing when investigating MDV miRNA function is worthwhile, as such a cellular mechanism has been demonstrated in another herpesvirus, human herpesvirus 6 (HHV-6), to disrupt mitochondrial architecture, evade intrinsic host defence mechanisms, and facilitate the transition from latent to lytic infection^74^. With the development of new bioinformatics technologies, other types of ncRNAs, including long noncoding RNAs (lncRNAs) and circular RNAs (circRNAs), have also been gradually identified and shown to play critical roles in MD biology^23^. Thus, the regulatory networks formed by ncRNAs in antiviral immunity also should be explored in the future.

Currently, the continued increase in the virulence of MDV has prompted ongoing efforts to develop more efficacious vaccines. Several techniques have been developed to selectively deliver protective antigens to APCs to enhance the protective efficacy of vaccines^127^. The recombinant viral vectors are also used for the development of novel poultry vaccines due to long-term antigen production and the delivery of antigens targeted with APC-specific antibodies. Several avian herpesviruses, such as HVT FC-126, infectious laryngotracheitis virus (ILTV) and some MDV-1 strains, have been genetically modified to be vectors for vaccines combating multiple avian diseases^128^. Recently, the use of extracellular vesicles (EVs) has emerged as a novel strategy for developing animal vaccines^129^. EVs have a potential role in the activation of cellular and antibody immune responses in MDV infection, thus opening up the possibility of developing a new MDV vaccination platform^130^. Considering the increasing number of important roles of the innate immune system in protection mediated by vaccination, the protective capacity of MD vaccines would greatly benefit from the development of new recombinant vaccines or adjuvants that enhance immune activation. More biological techniques, especially the generation of CRISPR/Cas9-mediated genome editing technologies^131^, must be channelled into the practical generation of novel, efficacious, safe, and broadly protective vaccines.

Considering that the currently used MD vaccines cannot prevent the infection and spread of MDV epidemic strains, additional alternative strategies for the efficient control of this disease are needed. In a recent study^132^, the sulfated polysaccharide extract ulvans from Ulva armoricana was verified to exert antiviral activity by limiting the replication/dissemination of viruses or stimulating the innate immune system of hosts, which may be an alternative to drug therapy for MDV infection. A study on the differential expression of purinergic receptors (PRs) in response to MDV infection and disease progression has demonstrated their crucial role in the process of disease; PRs may provide another potential target for MD therapy^133^. The generation of breeding chicken lines with more robust immune resistance to MDV may be another effective strategy to address the current epidemic and emerging viruses. Transgenic chickens stably expressing Cas9 and gRNAs to target the viral ICP4 gene for intracellular defence against MDV were recently generated to increase resistance to MD^134^. Furthermore, studies on the genetic variation in IFN signalling pathway genes may provide more possibilities for enhancing resistance to MD in chickens^135^.

In summary, we have reviewed the known strategies by which MDV evades immune surveillance mediated by distinct subsets of the host immune system. With the recent advancements in biotechnology and the application of new approaches in virology, more findings about the cellular immune response to MDV infection and/or vaccination will be uncovered in the near future. All of this meaningful progress will facilitate our work on the development of the next generation of efficient MD vaccines.

### Reporting summary

Further information on research design is available in the Nature Research Reporting Summary linked to this article.

### Supplementary information

Reporting Summary

## Data Availability

All data needed to evaluate the conclusions in the paper are present in the paper and/or in the materials cited herein. Additional data related to this paper may be requested from the authors.
